# A confidence predictor for logD using conformal regression and a support-vector machine

**DOI:** 10.1186/s13321-018-0271-1

**Published:** 2018-04-03

**Authors:** Maris Lapins, Staffan Arvidsson, Samuel Lampa, Arvid Berg, Wesley Schaal, Jonathan Alvarsson, Ola Spjuth

**Affiliations:** Department of Pharmaceutical Biosciences, Uppsala University, Box 591, 751 24 Uppsala, Sweden

**Keywords:** Conformal prediction, Machine learning, QSAR, Support-vector machine, LogD, RDF

## Abstract

Lipophilicity is a major determinant of ADMET properties and overall suitability of drug candidates. We have developed large-scale models to predict water–octanol distribution coefficient (logD) for chemical compounds, aiding drug discovery projects. Using ACD/logD data for 1.6 million compounds from the ChEMBL database, models are created and evaluated by a support-vector machine with a linear kernel using conformal prediction methodology, outputting prediction intervals at a specified confidence level. The resulting model shows a predictive ability of $\hbox {Q}^{2}=0.973$ and with the best performing nonconformity measure having median prediction interval of $\pm ~0.39$ log units at 80% confidence and $\pm ~0.60$ log units at 90% confidence. The model is available as an online service via an OpenAPI interface, a web page with a molecular editor, and we also publish predictive values at 90% confidence level for 91 M PubChem structures in RDF format for download and as an URI resolver service.
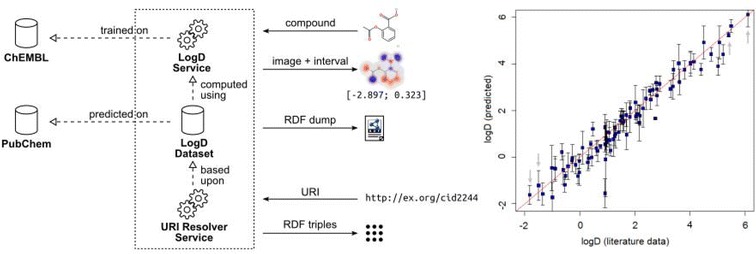

## Background

Lipophilicity plays a crucial role in determining the pharmacokinetic behavior of drugs. Hydrophilic compounds are typically well-soluble but are likely to exhibit problems with membrane permeability and are more susceptible to renal clearance. Highly lipophilic compounds tend to have low solubility, high plasma protein binding, and they are also more vulnerable to CYP450 metabolism. Furthermore, high lipophilicity has been shown to increase the likelihood of target promiscuity and general toxicity as well as more specific toxicology issues of hERG inhibition, phospholipidosis and CYP450 inhibition [1–3].

From these considerations, it is suggested that optimal ADME properties and the lowest risk for adverse toxicity outcomes are expected if a compound’s lipophilicity at $\hbox {pH}=7.4$ lies in a logD range between about 1 and 3 [2] or a logP between 2 and 4 [3]. Several studies indicate that these ranges might be even narrower depending on molecular weight, acid/base properties and/on the desired mode of action of the drug. For example, statistical analysis of AstraZeneca Caco-2 membrane permeability data suggests that the lower limit for passive diffusion is dependent on the molecular weight of compounds: a $\hbox {logD}>1.7$ being required for a 50% chance of high permeability for compounds with molecular weight above 350 Da, $\hbox {logD}>3.1$ for compounds with molecular weight above 400 Da, and $\hbox {logD}>4.5$ for compounds with MW above 500 Da [4].

Similarly, analysis of in-house data from Pfizer demonstrates that most of the compounds satisfying both cell permeability and in vitro clearance criteria fall into a logD range between 0 and 3 [5]. This study also suggests that higher molecular weight compounds are more constrained in the range of acceptable logD values; the top of optimum region (referred to as “golden triangle”) peaking to logD of about 1.5 at MW of 500 Da.

Several studies have found that logD or logP of above 3 gives rise to promiscuity and risk for adverse in vivo toxicological outcomes [4, 6, 7].

Furthermore, toxicological liabilities such as hERG inhibition depend on the acid/base properties of a drug, the risk being particularly high for lipophilic bases. For neutral drugs a 30% risk for problematically high levels of hERG inhibition is estimated at $\hbox {logD}=3.3$ whereas for basic compounds such risk arises already at $\hbox {logD}=1.4$ [8].

In a study on CNS drug-likeness, Wager [9] concludes that the most desirable lipophilicity for blood–brain barrier penetration is a $\hbox{logD} \le 2$. A logD above 4 is unlikely for a CNS drug.

Taken together, lipophilicity is one of the molecular properties to address in early stages of drug design, to increase chances of selection of compounds that would not fail in development because of poor ADMET characteristics.

Many computational methods to predict logP have been described. Benchmarking of 18 of these methods has shown reasonable results for many of them, with the root mean square error of prediction (RMSEP) for a Pfizer in-house dataset of 96,000 compounds being 0.95 log units for consensus logP and slightly above 1 log unit for best individual algorithms [10]. Prediction of logD is, however, more difficult, as it involves both estimation of logP and estimation of acid and base $\hbox {pK}_{\mathrm{a}}$ constants of the compounds, which may introduce further error. Nevertheless, AstraZeneca in-house algorithm AZlogD and the commercial ACD/logD algorithm of Advanced Chemistry Development, Inc. [11] on AstraZeneca an in-house dataset showed a very good $\hbox {RMSEP}=0.49$ for AZlogD and a reasonable $\hbox {RMSEP}=1.3$ for ACD/logD [4].

In this study, we present a support-vector machine (SVM) model based on data from 1.6 million compounds in ChEMBL database with logD annotations from the ACD/logD algorithm. The model was distributed as a Docker container and made available as a publicly available web service exposed with an OpenAPI definition. We evaluated the performance of the model and predicted 91 M compounds from the PubChem database, and made these data available in semantic web format (RDF) for download.

## Methods

### Data set

ChEMBL is an open, large-scale chemical database containing more than 1.7 million distinct compounds with bioactivity data extracted from the chemical literature and calculated molecular properties [12]. From ChEMBL version 23, we extracted all compounds having the calculated property acd_logd (calculated logD) at $\hbox {pH}=7.4$, resulting in 1,679,912 compounds. Standardization of chemical structures was performed by ambitcli version 3.0.2, which is part of AMBIT cheminformatics platform and relies on the CDK library [13–15].

Standardization was performed using default settings except for the option ‘splitfragments’ that was set to TRUE. In this way, salt and solvent components were filtered away. After standardization and removal of duplicates the data set consisted of 1,592,127 chemical compounds. To evaluate the predictive ability of the developed models, we set aside a test set comprising 100,000 compounds. To perform predictions on the developed model we downloaded 91,498,351 chemical compounds of PubChem database [16], which were standardized in the same way as the compounds from the ChEMBL database.

### LogP and logD

The most commonly used measure of lipophilicity is logP, the log of the partition coefficient of a neutral (non-ionized) molecule between two immiscible solvents, usually octanol and water. The distribution coefficient, logD, takes into account both the compound’s non-ionized and ionized forms and in the determination of logD the aqueous phase is adjusted to a specific pH. Most of the drugs and the majority of molecules under research for pharmaceutical purposes do contain ionizable groups, and therefore logD should be used preferentially over logP as the descriptor for lipophilicity, especially when looking at compounds that are likely to ionize in physiological media. Of a particular interest is the logD at $\hbox {pH}=7.4$ (the physiological pH of blood serum).

### Signature molecular descriptor

The compounds were encoded by the signature molecular descriptor [17], generated by CPSign [18]. A signature molecular descriptor constitutes a vector of occurrences of all atom signatures in the dataset, where an atom signature is a canonical representation of the atom’s environment (i.e., neighboring and next-to neighboring atoms). Signatures distinguish between different atom and bond types, as well as between aromatic and aliphatic atoms in the atom’s environment. Presence of the same atom signature in several compounds thus indicates that these compounds share identical 2D structural fragments. Atom signatures can be calculated up to a predefined height (i.e., the number of bonds to the neighboring and next-to neighboring atoms that the signature spans). We here calculated atom signatures of heights one, two and three, which is a set of heights good both for modeling as well as for visualization purposes [19, 20].

In total 1,068,830 different 2D structural fragments were found in the dataset. Of these, 675,996 fragments were present in at least two compounds, 251,278 in at least ten compounds, and 50,293 in at least one hundred compounds.

### QSPR modeling by SVM

To model the relationship of logD to the molecular descriptors, we used SVM, a machine learning algorithm that correlates independent variables to the dependent one by means of a linear or nonlinear kernel function. Kernel functions map the data into a high-dimensional space, where correlation is performed based on the structural risk minimization principle; i.e., aiming to increase the generalization ability of a model [21].

We elected to perform correlation by the linear kernel using signature molecular descriptors comprised of a vector of 1,068,830 integers. This choice was also supported by results of our earlier, large-scale modeling study, where a linear kernel performed on par with the nonlinear but required dramatically less computational resources [22].

SVM with linear kernel requires fine-tuning of two parameters to obtain an optimal model, namely, the error penalty parameter *cost* and tolerance of termination criterion *epsilon*. We found optimal *cost* and *epsilon* by performing grid search with cost values ranging from 0.1 to 10 and epsilon values from 0.1 to $10^{-5}$. SVM models were created by the LIBLINEAR software as accessed from CPSign [18, 23].

### Conformal prediction

In the conformal prediction framework, conventional single value predictions are complemented with measures of their confidence. In the case of regression, the conformal prediction algorithm outputs a prediction interval around the single prediction point [24]. In QSPR modeling, the size of the prediction interval is determined by some measure of dissimilarity (nonconformity measure) of the new chemical compound to the compounds used in the development of the prediction model. Thus, the compound that is “typical” for the data set would more likely be given a smaller interval than a compound being in a less explored area or outside the modeled chemical domain [25–27].

The size of intervals also depends on the desired confidence level (also called *validity*) which is defined as the ratio of compounds for which the true value falls within the prediction interval. Validity can thus range from 0 to 100%, where 0% means that none of the prediction intervals include the true value and 100% means that all of them include the true value.

For *inductive conformal prediction*, the training set is split into a *proper training set* and a *calibration set*. The proper training set is used for creating a prediction model and the calibration set is used for comparing new compounds to existing ones and to estimate sizes of intervals for a certain confidence level. The *inductive* setting means that split and training is performed once and all subsequent predictions are done by the same model; splitting is typically done in such a way that size of calibration set is smaller than the size of the proper training set [26].

**Fig. 1:**
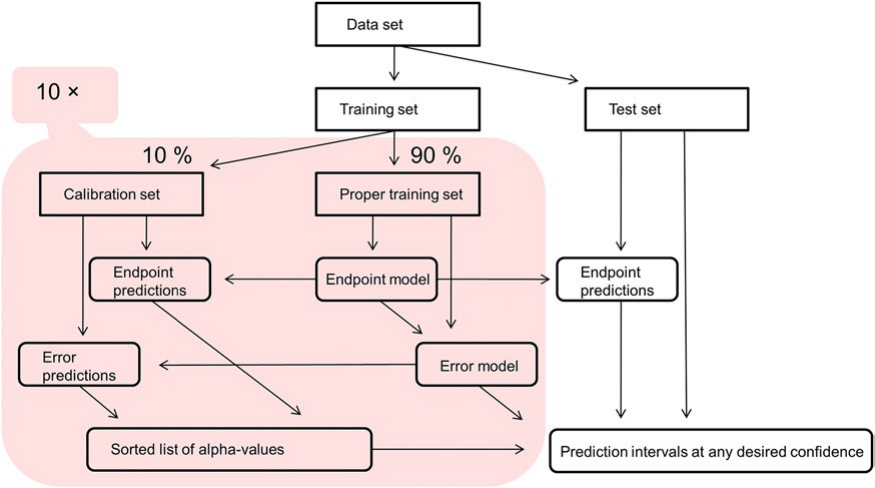
Workflow of 10-fold cross-conformal predictor. The training set is randomly permuted and split into ten, non-overlapping folds. An inductive conformal predictor (pink area) is trained for each split, using a single fold as its *calibration set* and the remaining nine folds as its *proper training set*. Proper training sets are used for fitting the endpoint and error models. Calibration sets are used to evaluate predictive ability of the model and to accumulate a list of $\alpha$ (compound nonconformity) values. For any new prediction, each inductive predictor will give an endpoint prediction (single-value prediction) and produce a prediction interval based on the predicted error, the desired confidence and the list of $\alpha$ values. The final prediction is computed by aggregating the individual predictions using the median midpoint and median interval width

In the present study, we applied a 10-fold *cross-conformal predictor* (CCP) as described in [28]. In brief, this algorithm attempts to reduce the influence of the split into proper and calibration sets by performing multiple such splits, each resulting in an inductive conformal predictor, and aggregating the resulting predictions. Here we chose to use ten aggregated models, and performing the dataset splits in a folded fashion (the *cross* prefix refers to *k*-fold cross validation). The workflow of CCP is presented in Fig. 1.

Conformal predictors are always valid under the assumption of exchangeability, i.e., that predicted compounds are drawn from the same distribution as compounds used to develop the prediction model. The main criterion when comparing different nonconformity measures is therefore their *efficiency*, i.e., the sizes of prediction intervals in case of regression. Intuitively, a smaller size of prediction intervals indicates a higher efficiency. In this work we evaluated three different nonconformity measures. The simplest measure tested here was based on the prediction error given by the endpoint model, where the nonconformity of compound *i*, denoted $\alpha _{\mathrm{i}}$ is calculated using Eq. 1. This measure, termed *absolute difference*, gives the same prediction interval size for all predictions for a given confidence level, but in turn does not require any error model to be fitted and can thus lessen the computational demands.

1 $$\begin{aligned} \alpha _{\mathrm{i}}=|y_{\mathrm{i}}-\hat{y}_{\mathrm{i}}| \end{aligned}$$

The second nonconformity measure used, termed *normalized*, assigns larger prediction intervals to objects that are more different from objects used in the model development and hence are “harder” to predict, and smaller intervals to “easier” objects. Naturally, when using normalized nonconformity measures, we expect the median prediction interval to be smaller, i.e., the efficiency to be increased. One of the common ways to obtain a normalized nonconformity measure is by creating an error model, where the dependent variable is the absolute value of error in the endpoint prediction model. This is expected to provide a more efficient nonconformity measure than absolute difference, provided that the error model is predictive. The normalized nonconformity measure is defined following Eq. 5 in [26], here shown in Eq. 2, where $|y_{\mathrm{i}}-\hat{y}_{\mathrm{i}}|$ is the absolute value of error for object *i* in the endpoint prediction model and $\hat{\mu }_{\mathrm{i}}$ is the prediction from an error model (note that both $\hat{y}_{\mathrm{i}}$ and $\hat{\mu }_{\mathrm{i}}$ are calculated when the compound is placed in the calibration set, i.e., is not present in the proper training set).

2 $$\begin{aligned} \alpha _{\mathrm{i}}=\frac{|y_{\mathrm{i}}-\hat{y}_{\mathrm{i}}|}{\hat{\mu }_{\mathrm{i}}} \end{aligned}$$

The third nonconformity measure, termed *log-normalized*, proposed in [25], instead of $|y_{\mathrm{i}}-\hat{y}_{\mathrm{i}}|$ uses $\ln {|y_{\mathrm{i}}-\hat{y}_{\mathrm{i}}|}$ as dependent variable when fitting the error model. It also introduces a smoothing factor, $\beta$, that can be used for “smoothing” the interval sizes, making the small intervals a bit larger and the very large intervals a bit smaller, i.e., reducing the influence of $\hat{\mu }_{\mathrm{i}}$ in calculating $\alpha _{\mathrm{i}}$, Eq. 3. The smoothing might be advantageous as biological measurements always include some measurement errors, precluding predictions with intervals close to 0. Very large intervals, on the other hand, can arise from badly predicted $\hat{\mu }$ in the error model. We here created models with $\beta =0$ and $\beta =1$.

3 $$\begin{aligned} \alpha _{\mathrm{i}}=\frac{|y_{\mathrm{i}}-\hat{y}_{\mathrm{i}}|}{e^{\hat{\mu }_{\mathrm{i}}} + \beta }; \quad \beta \ge 0 \end{aligned}$$

For each of the inductive conformal predictors, $\alpha _{\mathrm{i}}$ values are computed for all compounds in the calibration set and are then sorted in ascending order. When performing a prediction, the test compound is first predicted by the endpoint model to get the prediction midpoint, $\hat{y}$. To compute the prediction interval, the algorithm looks in the ordered set of nonconformity values to to get $\alpha _{\mathrm{conf. lev.}}$, which is dependent on the desired confidence of the prediction. If, for example, we propose that an 80% confidence is required, the $\alpha _{\mathrm{conf. lev.}}$ is then the $\alpha _{\mathrm{i}}$ value found when traversing 80% of the list. If the nonconformity value is dependent on an error model, an error prediction, $\mu _{\mathrm{i}}$, is made. The size of the prediction interval is then calculated by rearranging the nonconformity measure to solve for $|y-\hat{y}|$, resulting in the final prediction interval $(\hat{y} - |y-\hat{y}|, \hat{y} + |y-\hat{y}|)$ for the single inductive predictor. The CCP prediction is then computed to be the median prediction midpoint and the median predicted interval size.

### Molecule gradient for the prediction

CPSign allows the computation of a “prediction gradient”, as described in [29]. This is managed by altering the number of occurrences of each signature descriptor of the molecule, changing one descriptor at a time. For each alteration a new prediction is made, and the relative change in the prediction output is considered the gradient for that signature descriptor. If the gradient value for the descriptor is positive, the altered prediction has given a larger regression value, meaning that adding more of this descriptor would move the prediction to a higher response value, and vice-versa if the gradient value is negative. In CCP, each of the ten models produces its own gradient. The resulting gradient is computed as the median of the individual gradients. The *per-descriptor* contributions can then be transformed to the *per-atom* contribution, by summing up all contributions that each atom is part of.

## Results and discussion

### Development of CCP model

The data set was randomly split into a training set comprising 1,492,127 compounds and a test set comprising 100,000 compounds. The training set was then used to develop SVM models and the test set was used to fine tune model parameters and assess their predictive performance. Optimal model parameters were found by a grid search, starting with a low-complexity model with a low cost for errors, ${\textit{cost}}=0.001$, and a high tolerance for termination criterion, ${\textit{epsilon}}=0.1$. Note that the time required for model development and the model complexity increase along with higher *cost* and lower *epsilon* value. A too low value of *cost* and/or too high value of *epsilon* generally results in underfit models with low predictive ability. On the other hand, excessive *cost* and/or insufficient *epsilon* not only make the computations overly time-consuming but also gives rise to a risk for overfitting, indicated by decreasing training set errors but suboptimal predictive performance. As could be expected, the initial model showed low predictive ability, the squared correlation coefficient between acd_logd values of test set compounds and the predicted values being $\hbox {Q}^{2}=0.501$. The highest predictive ability of $\hbox {Q}^{2}=0.973$ was reached for model with ${\textit{cost}}=1$ and ${\textit{epsilon}}=10^{-4}$ (Table 1). As shown in the table, reducing *epsilon* (i.e., enabling more thorough model development) leads to major increase of predictive ability, whereas the influence of *cost* (penalizing large errors) is rather small at any *epsilon* level.

**Table 1:** Predictive ability of models, expressed as squared correlation coefficient ($\hbox {Q}^{2}$) between acd_logd values in ChEMBL database and predicted logD values for 100,000 test set compounds

Epsilon	Cost				
	0.001	0.01	0.1	1	10
$10^{-1}$	0.509	0.509	0.510		
$10^{-2}$	0.820	0.821	0.821	0.821	
$10^{-3}$	0.918	0.943	0.949	0.952	0.952
$10^{-4}$	0.923	0.958	0.970	***0***.***973***	***0***.***973***
$10^{-5}$		0.958	0.971	***0***.***973***	0.972

Bolditalic values indicate models with the highest predictive ability

**Fig. 2:**
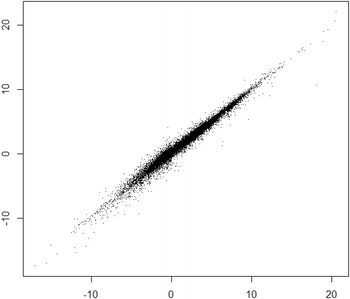
Predictive ability of the best model $(\hbox {Q}^{2}=0.973)$. Plotted are acd_logd values (x-axis) versus the predicted logD values (y-axis) for 100,000 test set compounds. The root mean square error of prediction (RMSEP) is 0.41 log units

Prediction results are illustrated graphically in Fig. 2, showing good correlation over the whole range of logD values.

After finding optimal settings for the logD model, we developed CCP models with *absolute difference*, *normalized*, and *log-normalized* (with $\beta =0$ and $\beta =1$) nonconformity measures. We elected to elaborate these models at three *epsilon* levels starting from 0.001. CPSign error models are necessarily created with the same settings as the endpoint model. However, intuitively it seems that the task of the error model of explaining mispredictions of logD model is quite difficult, taking into account that $\hbox {RMSEP}=0.41$ is already comparable to errors in experimental determinations of logD. Accordingly, the error model can be expected to be less predictive and more prone to overfit than the logD model.

The efficiency of the twelve developed CCP models are presented in Table 2. By comparing models based on *absolute difference* and the *normalized* nonconformity measure, it is apparent that the later ones are superior at certain ranges of confidence levels—from 50 to 99% when *epsilon* is 0.001, and from 70 to 90% when *epsilon* is $10^{-4}$. However, the *normalized* model does not outperform the *absolute difference* model when *epsilon* is $10^{-5}$. This finding confirms the assumption that error models may become overfitted if *epsilon* is very nonrestrictive.

**Table 2:** Median prediction interval width at confidence levels from 10 to 99%

Epsilon	Nonconformity measure	Confidence level										
		10%	20%	30%	40%	50%	60%	70%	80%	90%	95%	99%
$10^{-3}$	Abs-diff	0.109	0.221	0.336	0.462	0.604	0.771	0.986	1.284	1.813	2.237	3.841
	Normalized	0.122	0.243	0.362	0.478	0.595	*0*.*718*	*0*.*854*	*1*.*027*	*1*.*319*	***1***.***649***	***2***.***892***
	Log-normalized, $\beta =0$	*0*.*071*	0.155	0.257	0.387	0.560	0.801	1.171	1.812	3.291	5.273	10.879
	Log-normalized, $\beta =1$	0.074	0.159	0.260	*0*.*384*	*0*.*545*	0.763	1.080	1.599	2.689	4.031	7.676
$10^{-4}$	Abs-diff	0.069	0.139	0.211	0.288	0.378	*0*.*486*	0.629	0.843	1.245	*1*.*695*	*3*.*006*
	Normalized	0.079	0.157	0.233	0.311	0.395	0.491	*0*.*610*	***0***.***789***	***1***.***200***	1.918	7.194
	Log-normalized, $\beta =0$	*0*.*042*	*0*.*094*	*0*.*159*	*0*.*243*	*0*.*352*	0.519	0.772	1.223	2.311	3.918	10.157
	Log-normalized, $\beta =1$	0.044	0.097	0.163	0.245	0.356	0.509	0.741	1.137	2.030	3.233	7.204
$10^{-5}$	Abs-diff	0.065	0.132	0.201	0.270	0.354	***0***.***459***	***0***.***600***	*0*.*813*	*1*.*217*	*1*.*680*	*3*.*024*
	Normalized	0.075	0.148	0.220	0.293	0.376	0.474	0.605	0.824	1.445	2.664	12.199
	Log-normalized, $\beta =0$	***0***.***041***	***0***.***092***	***0.155***	***0***.***234***	0.341	0.495	0.738	1.171	2.205	3.747	10.007
	Log-normalized, $\beta =1$	0.042	0.095	0.158	0.235	***0***.***339***	0.486	0.710	1.095	1.963	3.156	7.247

Shown are MPI at confidence levels (validity) from 10 to 99%. Note that a smaller median prediction interval indicates higher efficiency of a nonconformity measure. Shown are results for models with ${\textit{cost}}=1$ and *epsilon* values $10^{-3}$, $10^{-4}$ and $10^{-5}$. Italicized are results for the best model at each epsilon value and confidence level. Marked by bolditalics are results for overall best models at each confidence level

Another result revealed by Table 2 is the very wide prediction intervals for *normalized* and *log-normalized* models at confidence level 99%, indicating that error model based approaches are not of practical use if one wants to achieve such a high confidence level. A somewhat surprising finding is that for low confidence levels (up to 50%) *log-normalized* nonconformity measure based models outperform all other models, being however less efficient at higher confidence levels. For example, if one could be satisfied with 20% confidence, then the median predictions interval width would be below 0.1 log units. Predictions at such a low confidence, however, does not seem to be of any practical use.

The overall-best model at any confidence level is in Table 2 indicated by bolditalics. In most practical CP studies, the desired confidence level is in the range of 80–90% [26, 27, 30–32]. Accordingly, for the prediction service we have selected a model that is most efficient for this range, and in fact, also shows very good efficiency at any other confidence level under 99%.

### Service for logD prediction

The logD prediction model with *normalized* nonconformity measure is available as a REST service using Swagger UI at: https://cplogd.service.pharmb.io/. Swagger [33] is a framework for making RESTful web based APIs available. It provides a standard for documentation, code generation as well as the Swagger UI, which is a web based interface where the endpoints of the API can be tested. The logD prediction model is made available with two endpoints:

- /prediction provides a prediction for a given SMILES at a user selected confidence level.
- /predictionImage provides images showing molecule gradient for the prediction.

Using the swagger service and the free molecule editor JSME [34], we also created a web-based user interface where a prediction image is rendered continuously as a molecule is edited (http://predict-cplogd.os.pharmb.io/ [35]). The user interface also supports selecting a confidence level using a slider which will render the prediction interval. Pulling the slider thus gives immediate response on the confidence effect on the prediction interval.

### Application of the logD prediction

We will here exemplify prediction results using two reference datasets of experimentally determined logD data.

The 29 compounds selected by Low et al. [36] represent those typically encountered in drug discovery programs, with MW up to 530 and polar surface area up to 114 Å$^{2}$. A set of 72 compounds collected by Alelynas et al. [37] shows a broader chemical diversity and range of logD values. In both studies, the literature data is used to validate results of newly-developed methods of logD measurement and the correlation is reported as $\hbox {R}^{2}$ of 0.982 and 0.997, respectively, which confirms accuracy of the data. Notably, in ten cases, there is a disagreement of more than one log unit between values reported in [37] and ACD/LogD calculation results, which indicates that affording accurate logD predictions and narrow prediction intervals for this dataset is a challenging task.

**Fig. 3:**
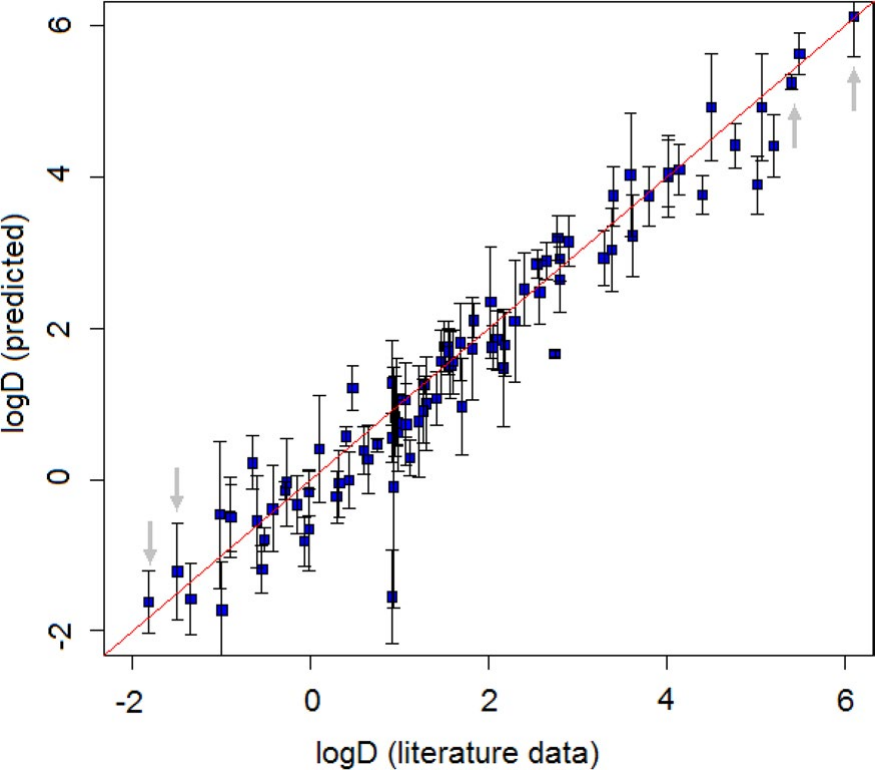
Example of prediction intervals. Shown are intervals at 80% confidence level for 29 reference compounds from [36] and 72 compounds from [37]. Grey arrows mark the compounds exemplified in Fig. 4

The prediction results at 80% confidence level are presented graphically in Fig. 3. The prediction is considered correct if the interval includes the true value (i.e., crosses the red-colored identity line). Note the variation in widths of prediction intervals, which for most compounds ranges 0.1–0.8 log units. Among the depicted set of compounds, the two widest intervals are given to strychnine ($\hbox {logD}=0.93$; prediction midpoint $-0.10$ and interval from $-1.70$ to 1.49) and furosemide ($\hbox {logD}=-1.02$; prediction midpoint $-0.46$ and interval from $-1.44$ to 0.51). In both cases, the predictions are correct. If predictions were performed by *absolute difference* nonconformity measure, the size of interval for any compound would be 0.843 log units (see Table 2). In this case, prediction intervals for the two “hard to predict” compounds, strychnine and furosemide, would not include the true value.

**Fig. 4:**
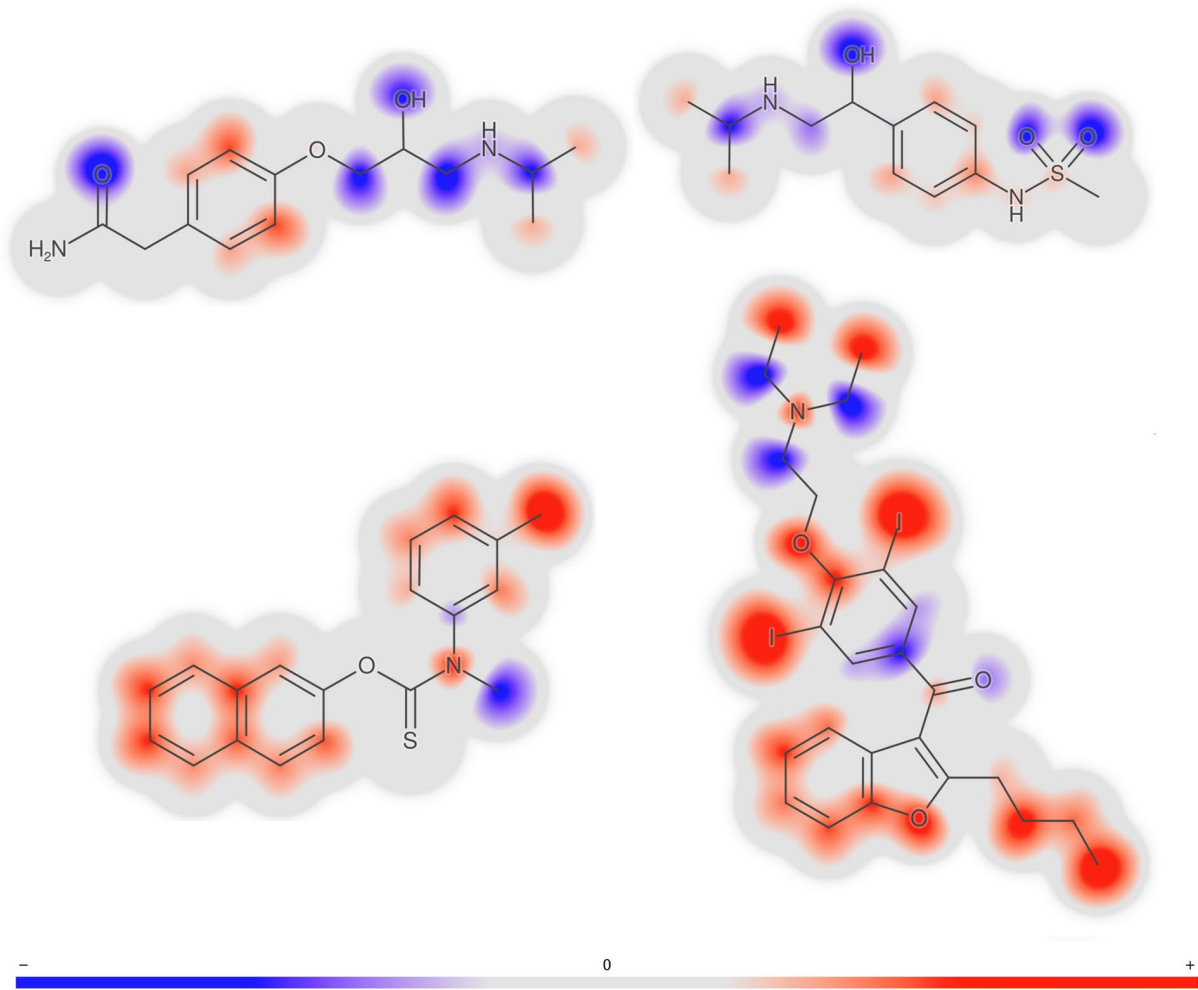
Examples of molecule gradients for the prediction of logD. Shown are gradients for four compounds indicated by arrows in Fig. 3. Upper row: atenolol ($\hbox {logD}=-\,1.82$) and sotalol ($\hbox {logD}=-\,1.52$). Lower row: tolnaftate ($\hbox {logD}=5.4$) and amiodarone ($\hbox {logD}=6.1$)

Molecule gradients for the prediction are illustrated in Fig. 4. The red-colored parts of the molecule contribute towards a prediction of higher logD and the blue parts contribute towards a prediction of lower logD. Note that the two hydrophilic compounds, atenolol and sotalol, are predominantly colored blue, except the phenyl rings that are colored light red and thus are predicted to increase lipophilicity. Note also that the propan-2-ylamino groups present in both compounds have similar but not exactly the same coloring. This is because each atoms is assessed in its environment of up to a three-bond distance (i.e., from all signatures of height one to three that include the given atom). In contrast to hydrophilic compounds at the top of the figure, the two highly lipophilic compounds at the bottom are predominantly colored red, except for the amine and ketone groups that are expected to decrease logD.

**Fig. 5:**
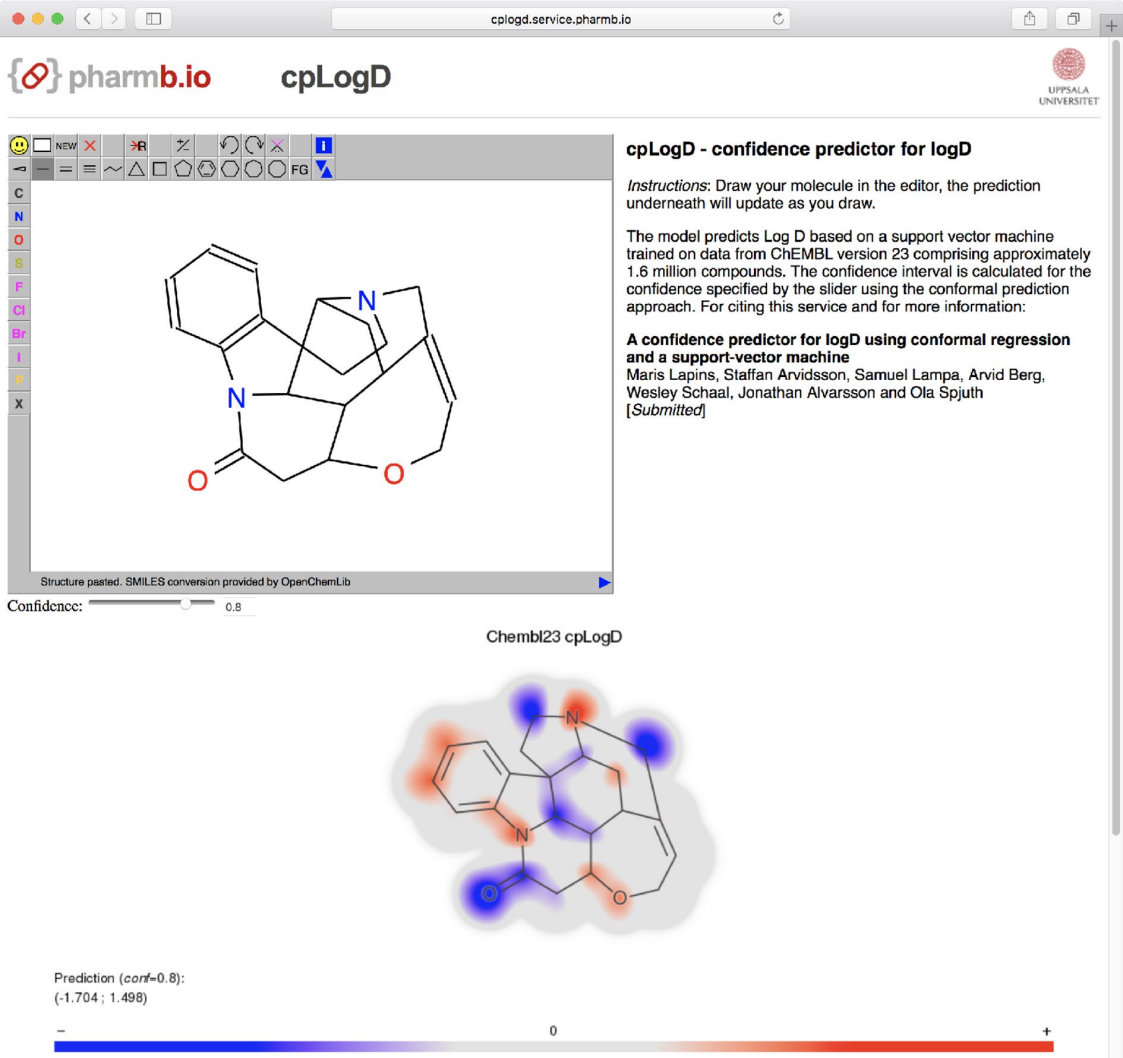
Example of a compound with large prediction interval as seen in the prediction service user interface. One compound which gives rise to a large prediction interval in Fig. 3 is strychnine ($\hbox {logD}=0.93$; prediction interval from $-1.704$ to 1.498). Here it is drawn using the prediction service available at http://predict-cplogd.os.pharmb.io/

Figure 5 illustrates a molecular gradient for a compound with a wide prediction interval, rendered in the user interface of prediction service at http://predict-cplogd.os.pharmb.io/. For this polycyclic alkaloid, the model has created quite a complex molecule gradient. In the upper panel of the interface a user can interactively modify molecule to inspect quantitative contribution of any modified atom(s) to the prediction of logD and to the width of the prediction interval.

### Dataset publication as RDF

The dataset of 91,498,351 compounds from PubChem with predicted logD values at 90% confidence level is in W3C RDF format [38]. It is available for download as data dumps in the Turtle RDF serialization format [39] and the indexed, binary RDF HDT format [40, 41] at https://doi.org/10.5281/zenodo.1091111 [42]. A URI resolver service is available at https://rdf.pharmb.io [43]. The URI resolver resolves the URIs of the new triples created for this dataset. It does so by providing all the triples linked to the resolved URI in N-Triples format [44] when accessing the URI via HTTP GET (the same as visiting the URL in a web browser). Newly created URIs for descriptors and compounds were minted off of the base URL of the URI resolver service. Annotation of descriptors is done using predicates from the Semantic Science Integrated Ontology [45]. Compounds are linked with *owl:sameAs* predicates to their corresponding URIs in the PubChem RDF service [46] data format. The data model provides descriptor nodes for each logD value from which the concrete values are linked. These descriptor nodes also contain other data, such as (OWL) class information. This allows the addition of further annotations and metadata either directly on the descriptor node or on its class node. For the URI publication service, a simple URI resolver software called *urisolve* was developed. The urisolve software resolves URI:s in a dataset based on an RDF HDT file or a SPARQL endpoint [47]. This software is available as open source at https://github.com/pharmbio/urisolve [48]. The urisolve software makes use of the RDF library for Go by Petter Goksøyr Åsen [49] for RDF serialization and the C++ HDT tools [50] for accessing the RDF HDT file.

## Conclusions

We have developed a confidence predictor for chemical compound lipophlicity (logD) using molecular signature descriptors and a support-vector machine. Unlike conventional regression, confidence predictor produces prediction intervals that satisfy a required confidence level. With normalized nonconformity measure, individual intervals are calculated for each compound. Model validation shows that the median prediction intervals ($\pm ~0.39$ log units at 80% confidence and $\pm ~0.60$ log units at 90% confidence) are tight enough to be useful in discovery.

The model is available as an online service via an OpenAPI interface and a web page with a molecular editor. Molecular signature descriptors allow interactive modification of molecules and visual interpretation of prediction results by highlighting chemical substructures contributing to the increase/decrease of the predicted logD.

We have also published predictive values at 90% confidence level for 91 million compounds of PubChem database in RDF format for download and as an URI resolver service.
